# An Automated Text Messaging System (Tonsil-Text-To-Me) to Improve Tonsillectomy Perioperative Experience: Exploratory Qualitative Usability and Feasibility Study

**DOI:** 10.2196/14601

**Published:** 2020-01-15

**Authors:** Nathan Farias, Benjamin Rose-Davis, Paul Hong, Lori Wozney

**Affiliations:** 1 Faculty of Medicine Dalhousie University Halifax, NS Canada; 2 Centre for Research in Family Health Izaak Walton Killam Health Centre Halifax, NS Canada; 3 Division of Otolaryngology Izaak Walton Killam Health Centre Halifax, NS Canada; 4 Division of Otolaryngology–Head & Neck Surgery Department of Surgery Dalhousie University Halifax, NS Canada; 5 Nova Scotia Health Authority Dartmouth, NS Canada

**Keywords:** short message service, tonsillectomy, pediatric otolaryngology, perioperative care

## Abstract

**Background:**

Inexperience and forgetting perioperative care instruction are significant drivers of parental stress during pediatric tonsillectomy care. With the widespread use of mobile technology, parents now desire a system that provides them with information that is timely, accessible, and comprehensive. Tonsil-Text-To-Me (TTTM) is a text messaging system that sends out automated and timed texts to parents of children who are undergoing tonsillectomy.

**Objective:**

The objective of this study was to pilot-test TTTM to assess for feasibility and usability and collect suggestions for system improvements desired by parents from a pediatric otolaryngology text message service.

**Methods:**

Parents of pediatric patients who were being scheduled for tonsillectomy with or without adenoidectomy were prospectively enrolled. An exploratory qualitative study using a semistructured interview guide was performed after parents received the automated texts 2 weeks before and 1 week after their child’s surgery.

**Results:**

A total of 7 parents were interviewed (data saturation was reached). Participants were all of maternal relation to the patient. Overall, all parents felt that the TTTM service was an improvement to the current standard model of information delivery. Parents also reported that the text messages reduced their anxiety and improved their performance when caring for their children during the perioperative period. No parents expressed privacy concerns about receiving texts and regarding the information included in the messages. Service suggestions showed that parents were eager for more information and had a high threshold for message reception regarding their child’s surgical care.

**Conclusions:**

All parents expressed enthusiasm for a text message service during their child’s tonsillectomy perioperative period. The care instructions and reminders provided to parents via automated and timed text messages may be a strategy to improve information delivery in a simple and accessible format that could empower families in their own health care.

## Introduction

Perioperative tonsillectomy care can be stressful for families. Parents often report that they would benefit from more information and direction. A family-centered approach requires the integration of high-quality resources to assist parents and improve adherence to posttonsillectomy protocols [1,2]. Traditionally, this has been accomplished with verbal, written, or printed discharge notes. However, as our society becomes increasingly media-driven, there is a need for technically focused medical resources that provide comprehensive information in a faster and more accessible format. With widespread use of mobile technology, most people now have access to a network-supported mobile device that provides short message service (SMS, also known as text messaging) [2]. These services provide a timely, effective, and financially viable platform that can facilitate convenient and comprehensive communication between health care services and patients [2].

Our team recently developed an automated text messaging service, Tonsil-Text-To-Me (TTTM), to expand our current perioperative consultation practices and assist parents in caring for their children. Prior to this study, our team completed a review of online recommendations for pediatric perioperative care following tonsillectomy and conducted a Delphi study with medical experts to develop an evidence-based list of recommendations for parents of children undergoing tonsillectomies [1,3]. The resulting data was used to generate our perioperative care–related text-messaging content for parents. We also completed the software development required to automatically and securely deliver SMS reminders to parents.

The purpose of this study was to pilot-test the implementation of TTTM into clinical practice and review feedback from parents using our service for the first time. The goal of the pilot test was to (1) obtain the opinions and suggestions of parents regarding their experience with the TTTM service and (2) confirm the software is functional and ensure an error-free SMS workflow for future clinic-wide implementation. Ultimately, field testing our SMS system will allow our team to assess its suitability and potential scalability into real-world clinical practice.

## Methods

### Study Design

Testing the usability and feasibility of interventions prior to full-scale testing and implementation can help to identify problems with acceptability and compliance and inform a full implementation strategy [4]. This exploratory usability and feasibility study used a qualitative semistructured interview guide to elicit the experience of parents who first used the TTTM SMS service. The qualitative methodology provided meaningful, in-depth feedback from relevant stakeholders and was informed by key usability and feasibility constructs explored in related research (eg, ease of use, satisfaction, learnability, safety, errors) [5]. Thematic analysis was performed on interview data and guided revisions to the format and content of TTTM.

Text messages were sent using email-to-text functionality from a designated institutional email so that it could be easily recognized by the participants. This setup also allowed for an audit trail through our email server. The SMS portal was built by our research team using Drupal, an institutionally approved software platform.

Ethical approval was obtained from the Izaak Walton Killam (IWK) Health Centre research ethics board (No 1021582).

### Recruitment and Eligibility Screening

Participants were parents of typically developing children (aged 3-14 years) who underwent tonsillectomy with or without adenoidectomy at the IWK Health Centre (a pediatric tertiary care hospital in eastern Canada). Inclusion criteria included parents’ age 18 years or older, fluent in English, have a cell phone, able to read text messages, and willing to receive 13 text messages over a 3-week period (2 weeks presurgery to 1 week postsurgery). Parents were excluded from the study if their child had complex medical needs beyond what is routinely accommodated for in tonsillectomy surgery such as previous history of peritonsillar abscess, complex chronic conditions, craniofacial abnormalities, diabetes, or a disorder in hemostasis.

### Sample Size

Previous studies investigating mobile health technology showed that preliminary usability issues can be detected with a sample size of 5 to 10 participants [6-8]. Thus, we aimed to recruit a minimum of 5 and continued recruitment until theoretical data saturation was reached [9,10]. After 7 interviews, no additional new insights were identified and therefore recruitment was stopped.

### Procedure

All eligible parents were offered service enrollment, in addition to conventional supports, during their child’s preoperative consultation. Information regarding our service was also advertised via posters displayed in the pediatric otolaryngology clinic and through otolaryngology clinic nurses. Recruitment began in January 2018 and ended in May 2018. Two months before their child’s surgery date, interested parents were contacted by a research team member, who further explained the study and obtained informed consent. Parents were offered a Can $20 (US $15) gift certificate (applicable to various retailers) as compensation for their involvement in our study. A nominated mobile number for contact during the study and the scheduled surgery date was recorded at the time of consent. Text messages delivered to parents during the study are presented in Table 1. Parents were contacted within 2 weeks of their last expected text message for their interview. Interviews were semistructured, audio recorded, and approximately 30 minutes in duration. All interviews were conducted by one researcher (NF) who did not have any previous relationship with the families. None of the families enrolled in our study were lost to follow-up.

**Table 1:** Tonsil-Text-To-Me text messages automatically delivered to parents during the perioperative period.

Perioperative period and text delivery day relative to surgery day		Text message
**Before surgery**		
	14 days before (morning)	Thanks for signing up for Tonsil-Text-To-Me! Your child’s surgery is coming up—time to get ready. Starting today stop giving your child aspirin.
	3 days before (evening)	You can help your child get ready for surgery. Be honest and up front about what will happen. Watch the day-surgery tour (URL).
	1 day before (evening)	Tomorrow is surgery day. Please stop giving solid foods 8 hours before, breast milk 4 hours before, and clear fluids 3 hours before surgery time. Learn more about how to manage pain after surgery (URL).
**Day of surgery**		
	Day of (morning)	It’s surgery day. We can do this! Bring a favorite toy to help your child feel more calm. Do you have everything you need? Day surgery checklist (URL).
	Day of (evening)	Surgery is over! You made it. Comforting your child will help them relax and relieve pain. Tips on how to comfort and distract them from pain (URL).
**After surgery**		
	1 day after (morning)	Check on your child after surgery for pain and breathing changes. Learn how to ask your child about pain (URL).
	1 day after (evening)	Eating soft foods and drinking clear fluids as soon as possible can help soothe your child’s throat. It’s okay for them to shower or bathe and brush their teeth as usual. Need soft food and clear fluid ideas (URL)?
	2 days after (morning)	Reasons to call your doctor: Bleeding in the nose or mouth Trouble breathing Your child seems dehydrated (should pee minimum 2x/day) or is refusing to drink fluids Pain that won’t go away, is getting worse, or isn’t helped with medication Fever greater than 39.0°C (102.2°F)
	2 days after (evening)	Remember, it is normal for a white coating to form on the tonsils as they are healing.
	3 days after (morning)	Have your child take it easy for the first 10 to 14 days after surgery (no sports, gym class, or roughhousing). No travel for 14 days.
	5 days after (morning)	It is normal for pain to peak around 5 to 7 days after surgery. Continue to give medication as directed to help your child get through this time.
	5 days after (evening)	Most children return to school after 7 days. When can they go back? Are they eating normally? Sleeping through the night? No longer need pain medication? If yes to all three, then school is okay.
	7 days after (morning)	Thanks for using Tonsil-Text-To-Me to help care for your child. This is your last message :)

### Data Analysis

Data analysis was initiated after the first interview. Interviews were transcribed verbatim and analyzed using thematic content analysis [11]. Participant responses were reviewed by the primary coder (NF) to identify an initial set of inductive codes. Themes and potential subthemes were noted. To minimize researcher bias, several meetings between the authors were held to review and refine coding scheme and approach to thematic analysis. In addition, a second researcher (LW) was appointed to review the transcripts using the refined codes to ensure emergent themes accurately represented the transcript data. The second researcher, who has experience in usability and feasibility research, found that the codes had content validity. All authors agreed that the themes were representative of the parent’s experiences with and feedback about the TTTM system.

Simple frequency analysis was conducted on quantified code data related to direct text recall from parents and text improvement suggestions. Frequency analysis was conducted using Excel (Microsoft Corp) and SPSS Statistics (IBM Corp).

## Results

### Participants

**Figure 1:**
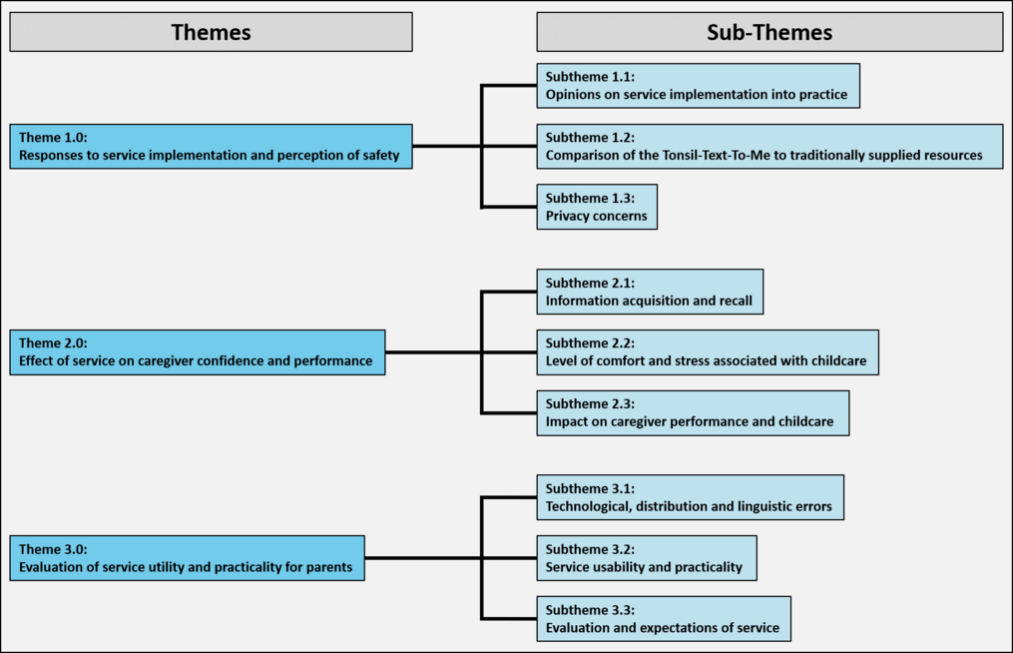
Themes and subthemes identified from the analysis of parental responses and feedback on the usability and feasibility of Tonsil-Text-To-Me.

After 7 interviews, there were no new insights obtained to inform design changes to the TTTM system and therefore recruitment was stopped. Participants were all of maternal relation to the patient. Three major themes were identified from parent interviews. These themes and their subthemes are shown in Figure 1 and described in detail below.

### Theme 1.0: Responses to Service Implementation and Perception of Safety

#### Subtheme 1.1: Opinions on Service Implementation Into Practice

Throughout the interviews, the response to TTTM was overwhelmingly positive. All families expressed encouraging remarks regarding TTTM service being implemented into practice.

#### Subtheme 1.2: Comparison of the Tonsil-Text-To-Me to Traditionally Supplied Resources

Parents commonly described having grievances with the current model of information delivery from health care providers. Mostly, parents reported forgetting, losing, or not having time to comprehensively review hard copy resources such as pamphlets or brochures.

I was frantically racing around the house the night before, trying to find the paper to figure out when she could eat last.

Participant 5

I know that’s all given in the active care pamphlet though, but if somebody lost that, maybe if it’s in the text they might not lose it.

Participant 4

It was a lot to take in, even though you had pamphlets and stuff.

Participant 2

The preoperative consult was another aspect of information delivery that was perceived as suboptimal. The combined effect of a large information load during consultation and lengthy gap between consultation and surgery date were cited as factors that disrupt information reception and overwhelmed parents.

During the consultation, which was months previous, a lot of the information was given to us by the nurses, and when you walk away you’re just like, oh, that’s a lot. But to have a text, it was almost more reassuring, and more of a comfort thing, if anything because it’s now closer to the time and you now can follow protocol, and not trying to remember everything from five months previous.

Participant 2

Of the three parents who had previous pediatric surgical experiences with their child, none reported being offered a service similar to TTTM. Two parents elaborated further to express that they felt TTTM implementation would be an improvement for parents compared to previous experiences. They appreciated how it was a useful tool that helped ease stress and enhance knowledge, especially for parents with no prior pediatric surgical experience.

Comparing it, I did really enjoy having this type of service...it’s comforting.

Participant 5

This was my child’s second surgery, so you know, I had different information going into this one than somebody who would be in the situation where it would be their child’s first surgery. So, I think it is definitely useful.

Participant 1

Although not explicitly asked, two parents self-reported that they found the URL links to additional information useful and time saving, as previously they would have to look for this information on their own. Overall, the ubiquitous enthusiasm for service implementation was in response to their desire for improved informative delivery in a simple and accessible format.

That I didn’t have to take my time to go online and research it. I could just actually look at the text and read the attachment and go, okay, oatmeal is good, because I actually was wondering that at one point.

Participant 2

I don’t think it gets much simpler than texting.

Participant 3

#### Subtheme 1.3: Privacy Concerns

When explicitly asked about comfort level and privacy concerns over receiving perioperative tips and reminders over SMS, none of the parents reported any such concerns. One parent commented that in terms of privacy, the content was perceived as a low-yield security risk, however acknowledging that others may feel differently.

Yes, I was comfortable getting them, and I was never concerned about privacy.

Participant 2

Yeah, I was comfortable and no, I’m not concerned about any of the privacy. It really doesn’t matter to me who knew that my child was getting surgery. But you know, other people may have those concerns.

Participant 1

In relation to the pediatric patients, all parents enrolled in the study were mothers, six of whom reported sharing the roll of perioperative care with other guardians (mainly fathers). Views over sending concurrent messages to other caregivers were mixed. Three parents expressed an interest in concurrently sent messages, while the other four parents were content receiving the messages themselves and relaying them to other guardians.

Yeah, I guess it could have gone to my husband as well. We share parental duties, I don’t think that it going to multiple numbers in the same household would be an annoyance.

Participant 1

### Theme 2.0: Effect of Service on Caregiver Confidence and Performance

#### Subtheme 2.1: Information Acquisition and Recall

The most commonly cited concern by parents was their propensity to forget or overlook care instructions.

I thought it was a good idea because parents are busy, and it’s easy to forget things. A simple text as a reminder of what needs to be done and when was nice.

Participant 4

I thought it was great, even though I knew it was coming up, I forgot kind of the different steps, like not giving her different medicines.... So, it was nice to have that reminder.

Participant 3

Parents reported that the service reminders facilitated an improvement in knowledge and helped them prepare for upcoming benchmarks in their child’s care.

One thing actually that did help me was a reminder to have pain medication, and the text was what prompted me to go buy that.

Participant 6

In the sense that if your child is being particularly distraught and upset, it’s nice to see a little message saying that it will end.

Participant 3

I found it helpful, and it definitely relieved stress in the fact that I knew that I was going to get messages, rather than having to rely on my memory of what was coming next.

Participant 4

#### Subtheme 2.2: Level of Comfort and Stress Associated With Childcare

Parents reported that preparing for surgery and caring for their children after surgery were stressful experiences. However, with SMS reminders, parents reported that they felt more informed, were reassured about their child’s condition, and felt better equipped to care for their child.

...when I read that I was like, oh, okay, now it makes me more aware of what he may be going through. Whereas before, if I didn’t get it I might be like, oh my god, what’s going on?

Participant 2

I think it would be a really good benefit, especially knowing just that you’re on the right track with it or just reminders to kind of keep you calm. Seeing your child go through a procedure is never easy, so it’s kind of just the peace of mind thing, as well as a nice reminder to make sure you’re still on the right track...

Participant 5

I mean, that was definitely a good reminder so that I could really lessen the feeling of his symptoms to what was supposed to be happening, so I didn’t panic and think that something was wrong when those symptoms occurred.

Participant 4

Also, despite knowing that the messages were being sent via an automated system, parents expressed feeling reinforced sense of support from their health care team.

It’s nerve racking leading up, and I’m stressed out leading up, so just kind of having that touch base check in type thing, even though I’m not actually speaking to anybody, it’s comforting.

Participant 5

If I had any stress, it would have taken it all away because it was like you had someone virtually guiding you through text about what to expect when you bring your child home after surgery.

Participant 2

Overall, parents reported that SMS reminders increased feelings of security and calm, prevented panic, and reduced stress when caring for their child.

I found it helpful, and it definitely relieved stress...through keeping the parent calm, and allowing the parent just to focus on one thing at a time, as opposed to stressing about everything all at once.

Participant 4

It was just that added security with regards to recovery, and all that stuff.

Participant 1

It was super helpful, instead of worrying and wondering; knowing that there’s going to be a text.... Yeah, it eased my mind a little bit, for sure.

Participant 7

Conversely, during prolonged intervals between subsequent SMS distributions, some parents reported increased stress levels, transiently. These feelings generally occurred during the preoperative period and were associated with ideas of abandonment by the service and not having accessible information to refer to.

At the ending, I felt it was really good, but in the beginning I was just waiting for another coming. [Interviewer: Did the beginning increase stress?] Yeah, I was expecting more information, for sure. I honestly felt like maybe you guys had forgotten about me.

Participant 7

And like I said, if the texts came once a day, it makes a parent feel like they have that support on a daily basis.

Participant 1

#### Subtheme 2.3: Impact on Caregiver Performance and Childcare

All parents reported the service was helpful in assisting with their child’s care, and 5 parents believed it improved their child’s care. Ambiguity in how parents perceived the phrase “improvement in your child’s care” was observed.

I thought it was very helpful...I don’t think it affected his care.

Participant 2

I think that it definitely is, I don’t know if it really benefits the children. It does I guess, through keeping the parent calm, and allowing the parent just to focus on one thing at a time, as opposed to stressing about everything all at once. So, I guess it helps the child, but it’s more of a parent thing.

Participant 4

I don’t think you can improve on how a parent’s care—because when you talk about caring, you’re talking about how they love their child, because care comes with that. So, parents tend to go by instinct...I think it better informs them in how to appropriately care for their child during this time.

Participant 1

All but one parent believed that the service would be a helpful tool to improve how parents prepare their child for surgery. All parents believed that the service would improve how parents care for their child postoperatively.

So, I wouldn’t say it’s anything to prepare them for the surgery. I think that was limited, but it was very good for the guidelines after the fact.

Participant 2

I think it does assist them in preparing, like I got the text about stop giving, I believe it was aspirin or something like that. I believe it was a week or two before the surgery, so things like that, that parents may not even think about are beneficial. I think that it made me better able to deal with the situation and the care after, well during and after.

Participant 1

It was super helpful because I found afterwards they were bang on, when we would be having those sorts of questions. So, I thought it was super beneficial.

Participant 7

### Theme 3.0: Evaluation of Service Utility and Practicality for Parents

#### Subtheme 3.1: Technological, Distribution, and Linguistic Errors

No technical issues interfering with text reception such as a lost or stolen mobile phone, change in surgery date, or phone malfunction were reported. However, two parents reported that they received the final closing text message three consecutive times.

They were all fine. The only thing is, I think I got that last one about three times, so it might have been a little glitch.

Participant 6

The only thing I noticed was that the very last text saying that this would be the last one, I got three different times on three different days.

Participant 3

No grammatical or spelling errors were reported. The reading level of the messages was determined to be appropriate as no literacy barriers were recognized by parents.

### Subtheme 3.2: Service Usability and Practicality

Parents were prompted to comment on message quantity, length, and time of delivery. All parents were content with overall message lengths, stating that they provided a sufficient but manageable information load. Likewise, all parents were satisfied with the time of day that the messages were delivered.

I found everything straightforward. They came through at a reasonable time. It wasn’t like too early in the morning or too late at night. And they were short and sweet and to the point. I didn’t have to read paragraphs of information. It was very straightforward.

Participant 4

While no parents reported experiencing superfluous text reception, all but one reported a desire for more messages, particularly in the preoperative period. Some parents proposed message delivery frequencies as high as one message every 1 to 2 days as being optimal.

I think you could do it daily for frequency, and that even wouldn’t be a nuisance, just getting a text daily about things, changes, things like that is appropriate.

Participant 1

If you were to add more, it would just be to reiterate the information people have.

Participant 6

#### Subtheme 3.3: Evaluation and Expectations of Service

Parents were prompted to express their expectations and level of satisfaction with the service. Only two parents reported that the service completely satisfied expectations. Among parents who expressed the service fell short of their expectations, four reported that they expected more preoperative information and one anticipated receiving live message responses.

There was one text that I replied to, so I expected to get a reply back, so I guess that was my expectation—that if there were questions that I could ask them, and get an answer back, and that wasn’t the case.

Participant 1

I found the information before the surgery was minimal. And I guess I was thinking that more information would have been provided before the surgery. I find the information after was really, really good, and there was not an abundance of it.

Participant 2

I think it’s a super useful tool, just felt there could have been more information in the beginning.

Participant 7

**Figure 2:**
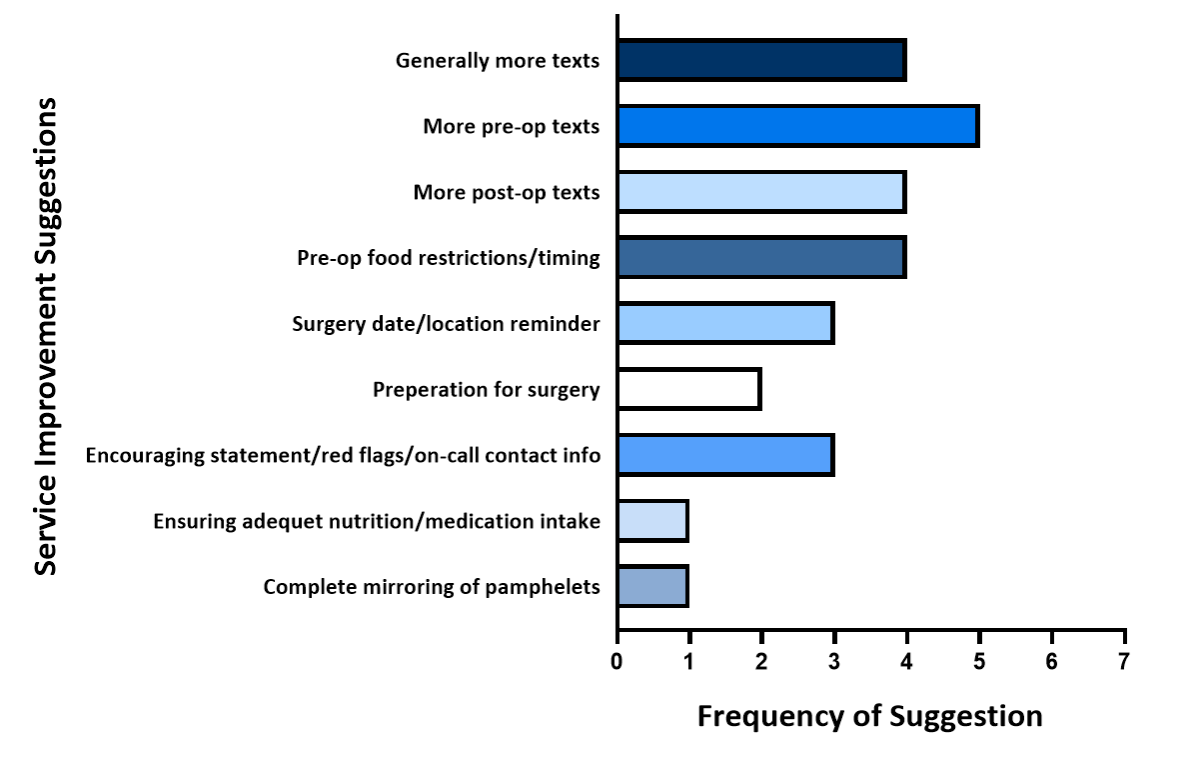
Frequency of every service improvement suggestion mentioned at least once throughout a participant interview.

The most frequent messages and information that parents recommended be incorporated into the TTTM service were recorded throughout the interview and quantified. They are summarized in Figure 2.

## Discussion

### Principal Findings

In this feasibility and usability study, parental response to SMS service implementation into our practice was strongly positive. Parents were particularly satisfied with the format of information delivery compared with traditional instructive practices such as verbal and written modalities. This supports data from Hofstetter et al [12], who studied vaccine reminder preferences and showed that parents much preferred SMS reminders over phone calls from clinic staff, written reminders, or automated phone reminders. Text messages are favored because they are timely, brief, and to the point. Additionally, they have the added advantage of integrated links to other forms of media, such as websites and videos, which helps personalize care to individual parental needs. All parents were comfortable receiving text messages on their phones and had a very low level of concern regarding the information they received via SMS. However, to safeguard patient confidentiality, TTTM reminders excluded patient names and any identifiable information. Thus, these safety findings may not be readily extrapolated to other platforms.

Parents who had previous surgical experiences with their child reported no similar mobile device reminder tools or accessible online services offered to them prior to TTTM. This highlights a major area of need for providers. In the absence of health care team–derived platforms to deliver tailored information, the internet has become the resource of choice for parents seeking more information about their child’s condition [13,14]. Pehora et al [15] showed that following day surgery, 98% of parents used internet search engines to find information regarding their child’s health, despite only 24% reporting that they regarded this information as reliable and safe. The high, yet reluctant, use of such poor resources illustrates the anxiety and helplessness that parents feel when having to self-sufficiently manage their child’s perioperative care needs without adequate resources. The combination of a society that highly desires information in an online or mobile device format with the availability of misinformation online should warrant concern in health care. Thus we believe that SMS is a reliable and inexpensive method to deliver clinician-reviewed instructions with links to trusted resources to parents.

Parent perception of benefit, convenience, and integration into daily life is essential for successful service implementation. In this study, incorrectly recalling perioperative care instructions comprised the bulk of parental anxiety when caring for their child, results that corroborate with previously published reports [16-19]. Participants in our study felt that information sent via SMS was an ideal strategy to help remember important care instructions. Timely delivery of pertinent information helped parents prepare for important milestones in their child’s care, such as organizing appropriate pain medication during the expected period of highest pain. While our study did not include objective measures of parental memory, a previous study by Yang et al [20] showed that mothers receiving reminder information via text messaging performed better on a knowledge assessment survey compared with mothers provided with the same information by conventional means (verbal and written form). This illustrates how SMS reminders can help parents be more informed, reassure them about their child’s condition, and make them better equipped to care for their child.

While parents clearly perceive the service as helpful to them, we have yet to determine whether perioperative text-messaging reminders make a significant impact on preoperative errors (such as appropriate fasting and timely arrival for their procedure) and postoperative outcomes (postoperative recovery and subsequent health care use). Studies suggest that uncertainty in knowing how to respond to the tasks of their child’s rehabilitation are associated with significant errors in care [16-19]. For instance, studies investigating postoperative pain management show that parents struggle to adequately assess their child’s pain and often provide less than optimal analgesic medication [21-23]. Consequently, uncontrolled pain subjects children to increased nausea, vomiting, and dehydration, which accounts for one-third of all posttonsillectomy emergency department use [24]. Reminders and information sent via SMS have the potential to improve child care perioperatively, reducing unnecessary health care use such as emergency department visits and clinic calls. The best evidence so far comes from a quality improvement study and pilot study, both by Newton and Sulman [25,26], who show that in a group of 85 parents receiving perioperative reminder text messages at their institution, none of them required procedure cancellation or postoperative emergency department visits. They also report reduced postoperative phone calls from parents (25%) compared with previous studies conducted without text-messaging reminders (29% to 40%) [25,26]. However, it is worth noting that these studies lacked control groups and thus no significance can be drawn from these results. A comparative trial of TTTM service is underway and will help illuminate these potential impacts.

In general, parents were not concerned with high text volumes and in fact most indicated that they anticipated more frequent messages, with some suggesting that daily texts would be acceptable. Sharifi et al [27] has reported similar results, showing that after receiving informative SMS messages on behavioral modification for pediatric obesity, parents sought more frequent text reception and even valued a mixture of instructive and reassuring content sent to them. Interestingly, despite knowing messages were sent via an automated system, parents reported that receiving regular texts made them feel continuously supported by their health care team. The parental perception of continuous support by the health care team is a novel insight into the value of perioperative SMS reminders to parents and is a functional outcome worth investigating for future studies.

### Limitations

Several limitations to our findings should be considered. Our study had a small sample size and other than relationship to the patient, parental demographic information was not collected. Thus, our results may not be representative of the entire eligible population, and we can not extrapolate these results to any general population other than those who are serviced by our institution. As well, the low number of participants may have limited the power of our study to reached thematic saturation for all possible factors associated with our SMS reminder system.

Our participants were all parents of children who received care at our institution. It is possible that existing client-provider relationships or experiences outside of this study could have biased interview responses. However, efforts were made to minimize potential impact. Participation was voluntary and parents could withdraw at any time; parents were explicitly made aware that participating in our study would have no impact in the care that they or their child received at our institution and that clinicians were blinded to identifying information of participants involved in the study. However, this study succeeded in providing real-world feasibility testing and collecting valuable feedback to improve the usability of our system. As a result, progress has been made on updating SMS reminder content, frequency, and regularity. Field testing our system also helped to identify a software malfunction associated with replicate messages that was subsequently investigated and mended. Currently, further testing is underway to gain insight into how large-scale rollout of our system will influence postoperative health care use and functional outcomes following pediatric tonsillectomy.

### Conclusion

This study specifically focused on the stakeholder perspectives to optimize adoption by parents and adequately address their needs with technological health information and resources. In doing so, we identified novel insights into parental preferences regarding text message reminders to support their child’s perioperative care and developed themes that can be used to guide future interventions. The key strategy for successful implementation was delivering comprehensive and relevant information at appropriate and regular intervals. Providing efficient and adaptable information to parents translated into confidence when caring for their children. Parental support for perioperative care instructions provided via SMS was strong and may be a cost-effective strategy to overcome recall errors, lessen parental anxiety, and empower families in their own health care.

## Abbreviations

IWK Health Centre: Izaak Walton Killam Health Centre
SMS: short message service
TTTM: Tonsil-Text-To-Me
